# Publication Volume and Equity in Competitive Surgical Residency Programs: A Bibliometric Analysis of the 2023-2024 Match

**DOI:** 10.7759/cureus.82451

**Published:** 2025-04-17

**Authors:** Garrett Dyess, Maxon Bassett, Noah Baker, Christian Cooper, Isabella Dinelli, Qays Aljabi, Ryan McIlwain, Joshua Baroody, Danner W Butler, Ben Sotello, Michael Rallo, Neil Goldsmith, Benjamin Dribbus, Harris Bolus, Charles Ogles, Ivy Nguyen, Jai Thakur, Nitin Agarwal, Phillip V Gordon, Richard P Menger

**Affiliations:** 1 Medical School, University of South Alabama College of Medicine, Mobile, USA; 2 Orthopedic Surgery, University of South Alabama College of Medicine, Mobile, USA; 3 Health Sciences, University of Alabama at Birmingham, Birmingham, USA; 4 Medical School, Rutgers Robert Wood Johnson Medical School, New Brunswick, USA; 5 Mathematics, William Carey University, Hattiesburg, USA; 6 Neurosurgery, University of South Alabama College of Medicine, Mobile, USA; 7 Neurological Surgery, University of Pittsburgh Medical Center, Pittsburgh, USA; 8 Neonatology, Infirmary Health, Mobile, USA; 9 Neurosurgery and Political Science, University of South Alabama College of Medicine, Mobile, USA

**Keywords:** bibliometric analysis, competitive specialties, medical education, nih funding, publication disparities, research productivity, residency match, usmle step 1

## Abstract

The “Charting Outcomes in the Match” report by the National Resident Matching Program compiles data on abstracts, presentations, and publications (APPs) but does not specify the number of peer-reviewed publications. Prior research indicates discrepancies between the number of APPs and peer-reviewed publications. This study aimed to characterize the drivers of heightened publication rates among applicants who match into competitive surgical residencies.A retrospective cohort comparison study was conducted from October 2023 to January 2024. Data were extracted using publicly available information. An institutional review board waiver was obtained from the University of South Alabama. All applicants in the United States who successfully matched in 2023 into orthopedic surgery, vascular surgery, otolaryngology, plastic surgery, or neurosurgery were investigated. Extracted data included matched specialty, sex, graduating from a top-40 National Institutes of Health (NIH)-funded medical school, attending residency at the same institution where one graduated, additional degree, and matching into a top-40 Doximity-ranked residency program. The primary outcome was the number of peer-reviewed publications by matched applicants in each specialty. Secondary outcomes assessed the impact of demographic factors on publication output, the distribution of top 10th percentile publishers by specialty, the proportion of individuals with zero publications, and the H-index of published applicants. Attending a top-40 NIH-funded school was associated with increased publication output, with odds ratios (ORs) of 3.31 (95% confidence interval (CI) = 2.14-4.47, p < 0.001). ORs represent the strength of association between predictor variables and publication metrics, with values above 1.0 indicating positive associations. This association was higher for neurosurgery (OR = 7.76, 95% CI = 5.55-9.76, p < 0.001) and plastic surgery (OR = 3.87, 95% CI = 1.98-7.77, p < 0.001). Conversely, matching into orthopedic surgery (OR = 1.09, 95% CI = 1.04-1.15, p < 0.001) or vascular surgery (OR = 1.07, 95% CI = 0.94-1.22, p < 0.001) predicted admission without having published a peer-reviewed paper. Neurosurgery applicants had a significantly higher H-index compared to other specialties. Neurosurgery and plastic surgery admitted applicants with more peer-reviewed publications and fewer with zero publications compared to orthopedic, vascular, and otolaryngologic programs. High publication rates were also predicted by holding a non-medical postgraduate degree and graduating from a top-40 NIH-funded medical school, raising equity concerns due to differences in research opportunities and additional degrees.

## Introduction and background

According to documents released by the National Resident Matching Program (NRMP) titled “Charting Outcomes in the Match,” the average number of research entries, defined as abstracts, presentations, and publications (APPs), has steadily increased over the past decade [1]. While every specialty has failed to see an increase in APPs over time, some specialties exhibit a greater rate of increase than others. Surgical specialties with marked publication increase include neurosurgery, plastic surgery, orthopedic surgery, otolaryngology, and vascular surgery [1,2].

Medical school enrollment has steadily grown over the past 20 years [3]. However, the number of residency training positions has not increased at the same rate [3]. While the total number of applicants entering the NRMP Match to total residency training positions has not exceeded total residency training positions, there are selected specialties with a surplus of applicants surpassing the available training positions [1]. The decreasing supply of residency positions per applicant in these selected specialties may contribute to the increasing research output, as the specialties with less supply seem to be the same ones experiencing rising rates of research output.

Major changes have occurred that likely influence the outcomes of the NRMP Match. Arguably, the most significant change is the United States Medical Licensing Exam (USMLE) Step 1 transitioning to a pass-fail grading system [4]. In response to these changes, a body of literature has emerged surveying program directors of competitive specialties to understand how weight will be re-distributed among features of residency applications, especially due to the USMLE Step 1’s shift to pass-fail. Survey data suggest that research output will be more heavily weighted as a result of this change, and bibliometric analysis is needed to analyze these changes as they unfold [5-9]. Understanding the distribution of publication output and its predictors is of interest to applicants, admissions committees, and NRMP personnel.

The primary objective of this research is to understand the drivers of peer-reviewed publications produced by applicants who have matched into highly competitive surgical residency programs, as defined by NRMP outcomes data. Secondary objectives include understanding predictors of research productivity across specialties that match applicants with high research output. It is important to note that this study is observational and does not imply causation between any individual factor and publication output.

## Review

Methodology

Data Collection

The number of APPs for each surgical specialty was extracted from the NRMP’s “Charting Outcomes of the Match” report from 2022. Specialties varied in their mean number of APPs, and three clusters of research output for surgical specialties were noted. Specialties with the greatest mean number of APPs included neurosurgery and plastic surgery. Orthopedic surgery, otolaryngology, and vascular surgery formed a secondary cluster with a lower mean number of APPs. Surgical specialties with a mean number of APPs less than six included general surgery and obstetrics and gynecology and were excluded from the dataset [2].

A list of residency programs meeting inclusion criteria (HB1) was obtained from the Doximity Residency Navigator website [10]. Each program was cataloged in a spreadsheet, and a research assistant used individual program websites to compile a complete list of individuals who matched into each program, resulting in a dataset of 1,710 matriculants.

Demographic data were collected using a three-tiered verification approach. The primary source was residency program websites, which provided most of the required data. When data were missing or unclear, we consulted secondary sources including LinkedIn and Twitter. A final verification layer involved reviewing peer-reviewed research articles authored by each applicant. The majority of program websites provided complete demographic data necessary for this study.

Demographic details recorded included matched specialty, sex, attendance at top-40 National Institutes of Health (NIH)-funded medical schools, presence of an Accreditation Council for Graduate Medical Education residency program in the matched specialty, possession of an additional degree, and matching into a top-40 research output program. Top-40 data for NIH funding and research output residency programs were sourced from the NIH’s RePORT and Doximity’s Residency Navigator, respectively [10,11].

Bibliometric data were obtained from Scopus by first gathering Scopus IDs using individual names and affiliations. Scopus IDs were collected for profiles found, with further searches conducted for individuals without IDs [12]. In cases where multiple profiles existed or names were common, searches were refined using institutional affiliation, known co-authors, and research topics to verify identity. Bibliometric data were then extracted from Scopus using a Python script to coordinate Scopus APIs; data included titles, publication dates, publishing journal names, citations, article type, institutional affiliations, and names of other authors for each publication, as well as total publications, H-index, and i10-index for each resident. Only peer-reviewed articles indexed by Scopus were included. Specialty-specific publications and basic science involvement were adjudicated based on the presence of search terms in each paper’s abstract as well as manual review. Two independent reviewers evaluated specialty-specific classifications. Discrepancies were resolved through discussion, and a third reviewer adjudicated any unresolved conflicts. Reporting guidelines for this study type are not readily available. The authors used guidelines from the BIBLIO checklist wherever possible to ensure rigor [13]. Data for this project can be made available upon request to the corresponding author.

Data collection was conducted from October 2023 to January 2024. Statistical analysis involved reporting categorical variables as counts and percentages, and continuous variables by measures of centrality (mean, median) and dispersion (standard deviation (SD), interquartile range (IQR)). Distribution assessments included visual inspections and the Shapiro-Wilk test. Publication percentiles were analyzed, and the impact of the individuals with the least research output and those with the most research output was calculated.

Univariate and multivariate analyses were used to control for variables such as NIH-funded school attendance, specialty matched, and additional degrees held. Models were used to determine the influence of these variables on publication output, never having published, and H-indices, once individuals who had never published were removed. No significant interaction effects were found. A p-value of less than 0.05 was deemed significant. All analyses were performed using R version 4.3.0 (R Studio, Vienna, Austria) and documented in Microsoft Word version 2022 (Microsoft Corp., Redmond, WA, USA) by the first author under the supervision of one of the senior authors.

Results

The majority of interns who matched at research-intensive surgical residency programs in 2023 were determined to be male (1,100, 64.33%), did not graduate from a medical school ranked in the top 40 for NIH funding (1,210, 70.76%), did not match into a residency program at the same institution they attended medical school at (1,261, 73.74%), did not possess an additional degree other than a medical degree (1,530, 89.47%), and did not match into a top 40 ranked residency program according to Doximity’s research output metric (1,237, 72.34%) (Table 1).

**Table 1:** Demographics and publication metrics of the 2023 matched surgical applicants. Available demographic information/comparisons of our 1,710 applicant dataset. NIH: National Institutes of Health; SD: standard deviation; IQR: interquartile range

Characteristics	Number (%)
Male	1,100 (64.33)
Female	610 (35.67)
Attended top-40 NIH-funded medical school	500 (29.24)
Did not attend top-40 NIH-funded medical school	1,210 (70.76)
Matched to home program	449 (26.26)
Matched to non-home program	1,261 (73.74)
Obtained additional degrees	180 (10.53)
Did not obtain additional degrees	1,530 (89.47)
Matched in top-40 residency program (Doximity Research Ranking)	473 (27.66)
Did not match top-40 residency program (Doximity Research Ranking)	1,237 (72.34)
Publications	Value(s)
Average (SD)	7.38 (11.39)
Median (IQR)	4 (9.75)
Range (Low to High)	0 to 113
Specialty-specific publications	Value(s)
Average (SD)	3.98 (7.6)
Median (IQR)	1 (5)
Range (low to high)	0 to 86
Basic science publications	Value(s)
Average (SD)	0.61 (2.28)
Median (IQR)	0 (0)
Range (low to high)	0 to 51
H-index	Value(s)
Average (SD)	1.9 (2.8)
Median (IQR)	1 (3)
Range (low to high)	0 to 31
I-10 index	Value(s)
Average (SD)	1.23 (3.22)
Median (IQR)	0 (1)
Range (low to high)	0 to 52

Distribution of Publication Output

The distribution of publication output was determined to be non-normally distributed and right-skewed (p < 0.001). Notably, 47.25% of the publications were produced by individuals in the top 10th percentile for publication output. However, 540 (31.58%) individuals in our dataset produced no publications. The median number of total publications in our dataset was 4 (IQR = 9.75), with a maximum of 113. The median number of specialty-specific publications was 1 (IQR = 5), with a maximum of 86. The median number of basic science publications was 0 (IQR = 0), with a maximum of 51. The median H-index was 1 (IQR = 3), with a maximum of 31, and the median I10-index was 0 (IQR = 1), with a maximum of 52 (Table 1).

Distribution of Publication Output by Specialties

**Figure 1:**
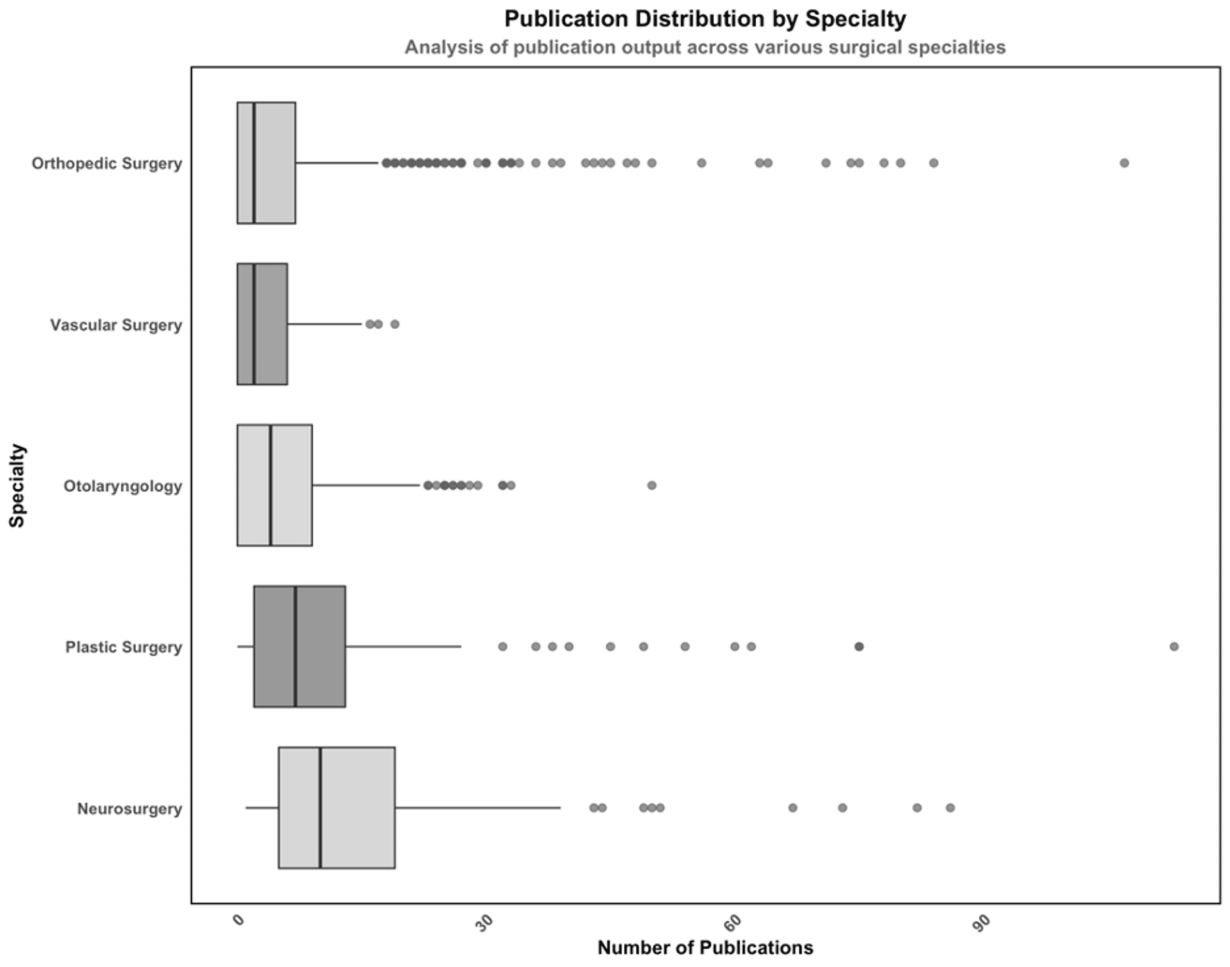
Boxplot of publication volume from applicants (x-axis) used to illustrate differences in medians, distributions, and outliers between surgical specialties (y-axis). Neurosurgery and plastic surgery have significantly more publications than their comparative residencies (p < 0.001 for each comparison).

Neurosurgery was found to have the highest median number of publications at 8, along with the greatest variation in publication output (IQR = 14). Following neurosurgery, plastic surgery had the second-highest median number of publications at 7 (IQR = 11). Orthopedic surgery and vascular surgery had the lowest median number of publications, each with 2 (IQR = 7 and IQR = 6, respectively) (Figure 1).

Multivariate analysis determined that matching into neurosurgery was the strongest predictor of publication volume (odds ratio (OR) = 7.66, 95% confidence interval (CI) = 5.55-9.76, p < 0.001), followed by plastic surgery (OR = 3.87, 95% CI = 1.98-5.77, p < 0.001). Possessing an additional degree beyond a medical degree (OR = 3.86, 95% CI = 2.11-5.62, p < 0.001) and graduating from a medical school ranked in the top 40 for NIH funding (OR = 3.31, 95% CI = 2.15-4.47, p < 0.001) were also found to be significant (Table 2).

**Table 2:** Multivariate and univariate predictors of publication output. Univariate and multivariate analysis of factors associated with increased publication volume support the relationships shown in Figure 1. *: significant p-value; NIH: National Institutes of Health; OR: odds ratio; CI: confidence interval

Variable	Univariate OR/β (95% CI)	P-value	Multivariate OR/β (95% CI)	P-value
Top-40 NIH-funded medical school	4.03 (2.86, 5.2)	<0.001*	3.31 (2.14, 4.47)	<0.001*
Home program	2.35 (1.13, 3.57)	<0.001*	-0.07 (-1.37, 1.23)	0.913
Additional degree	6.68 (4.95, 8.41)	<0.001*	3.86 (2.11, 5.62)	<0.001*
Specialty				
Orthopedic surgery	-0.16 (-1.49, 1.18)	<0.82	0.29 (-1.04, 1.62)	<0.67
Vascular surgery	-2.28 (-5.06, 0.5)	0.108	-1.76 (-4.54, 1.03)	<0.218
Otolaryngology	1.14 (-0.55, 2.01)	0.504	0.35 (-1.01, 1.03)	0.342
Plastic surgery	4.39 (2.49, 6.92)	<0.001*	3.87 (1.98, 5.77)	<0.001*
Neurosurgery	8.61 (6.68, 10.54)	<0.001*	7.66 (5.55, 9.76)	<0.001*

Predictors of Publication Output

**Figure 2:**
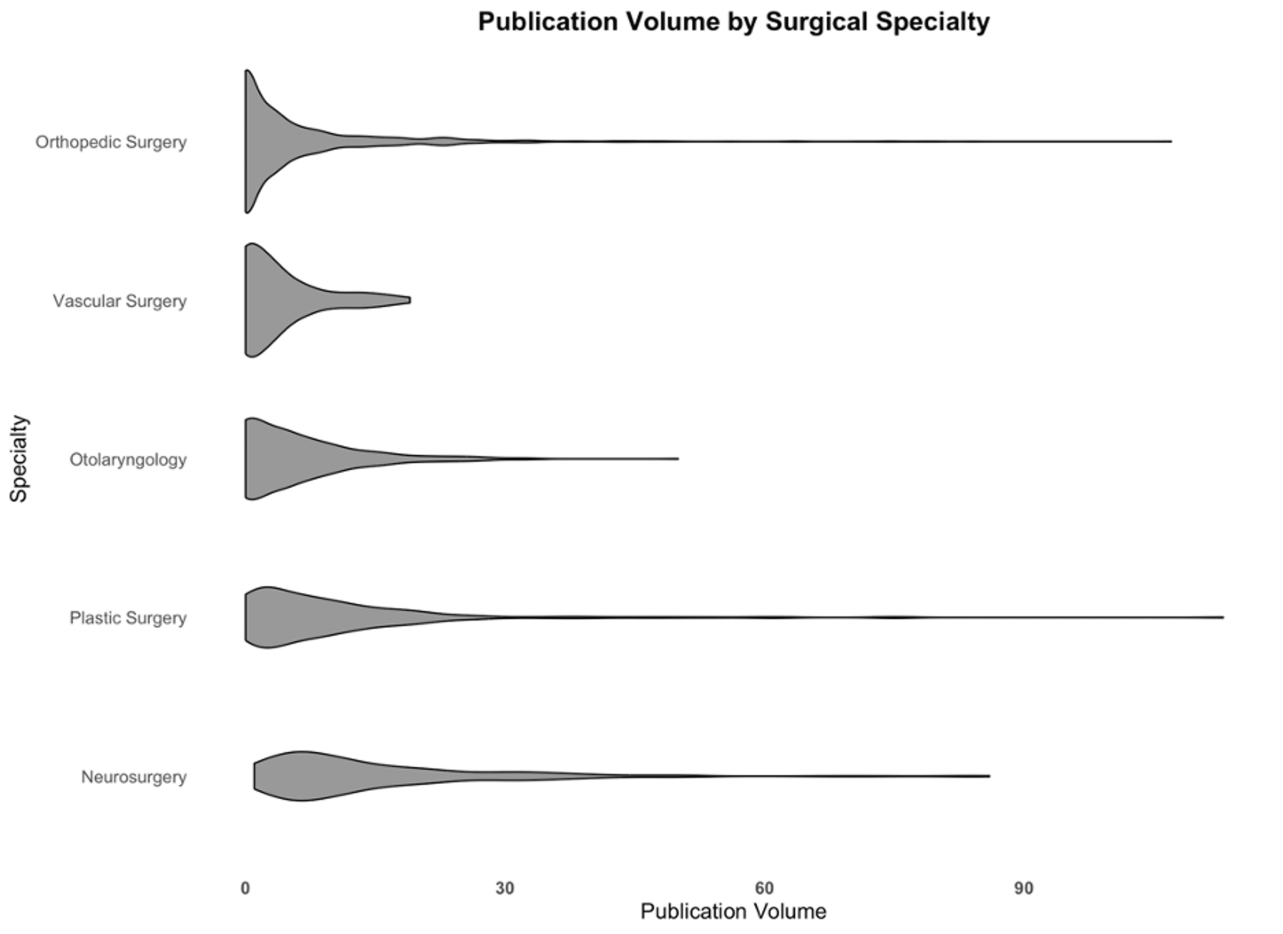
Violin plot of publication volume from applicants (x-axis) illustrating the differences in no or low publication applicants between surgical specialties (y-axis). Vascular surgery and orthopedics accept significantly more applicants who have never published than their comparative residencies (p < 0.001 for each comparison).

We grouped all applicants with zero publications and found that orthopedic surgery (43.2%) and vascular surgery (40.3%) had the highest percentages of these applicants. In contrast, neurosurgery (0%) and plastic surgery (6.1%) had the lowest percentages (Figure 2).

In multivariate analysis, attending a medical school ranked in the top 40 for NIH funding (OR = 0.82, 95% CI 0.79-0.86, p < 0.001) and matching into neurosurgery (OR = 0.75, 95% CI 0.69-0.82, p < 0.001) or plastic surgery (OR = 0.78, 95% CI 0.72-0.84, p < 0.001) were protective against never having published. Conversely, matching into orthopedic surgery (OR = 1.09, 95% CI 1.04-1.15, p < 0.001) was a predictor of never having published (Table 3). 

**Table 3:** Multivariate and univariate predictors of never publishing. Univariate and multivariate analysis of factors associated with surgical applicants who were accepted without a peer-reviewed publication support the relationships shown in Figure 2. *: significant p-value; NIH: National Institutes of Health; OR: odds ratio; CI: confidence interval

Variable	Univariate OR/β (95% CI)	P-value	Multivariate OR/β (95% CI)	P-value
Top-40 NIH-funded medical school	0.79 (0.75, 0.83)	<0.001*	0.82 (0.79, 0.86)	<0.001*
Home program	0.87 (0.83, 0.92)	<0.001*	0.97 (0.92, 1.01)	0.22
Additional degree	0.81 (0.75, 0.86)	<0.001*	0.95 (0.88, 1.01)	<0.11
Specialty				
Orthopedic surgery	1.12 (1.06, 1.18)	<0.001*	1.09 (1.04, 1.15)	<0.001*
Vascular surgery	1.09 (0.97, 1.22)	0.13	1.07 (0.94, 1.22)	0.26
Otolaryngology	0.91 (0.85, 0.94)	0.23	0.95 (0.84, 0.95)	0.32
Plastic surgery	0.77 (0.72, 0.83)	<0.001*	0.78 (0.72, 0.84)	0.24
Neurosurgery	0.73 (0.67, 0.79)	<0.001*	0.75 (0.69, 0.82)	<0.001*

Characteristics of the Top 10% Concerning Publications

**Figure 3:**
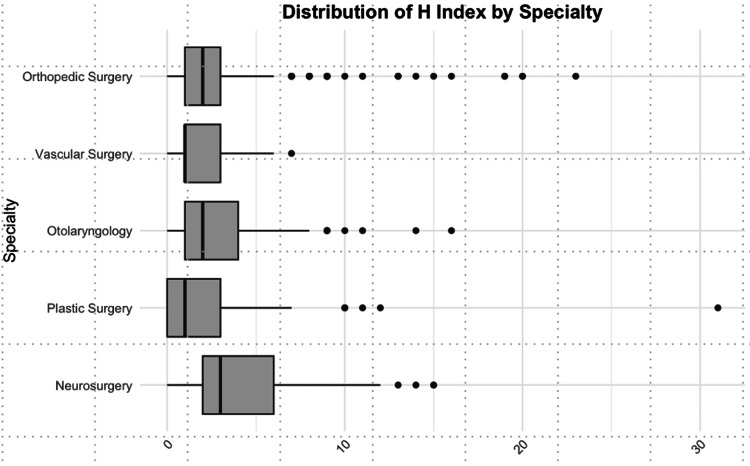
Boxplot of the index of applicants with applicants having never published removed (x-axis) illustrating differences in medians, distributions, and outliers between surgical specialties (y-axis). Neurosurgery has a significantly greater H-index than their comparative residencies (p < 0.001 for each comparison).

Subsequently, we calculated the median H-index across specialties and then removed applicants who had never published. Neurosurgery demonstrated the highest H-index across specialties in both conditions (p < 0.001). Plastic surgery, otolaryngology, and vascular surgery displayed a median H-index of 1, while orthopedic surgery had a median H-index of 0 when never-publishers were included. Upon removal of residents with no publications, the median H-index for otolaryngology and orthopedic surgery both increased to 2 (Figure 3).

Multivariate analysis indicated that attending a medical school ranked in the top 40 for NIH funding (OR = 2.31, 95% CI = 1.62-3.28, p < 0.001), acquiring an additional degree beyond a medical degree (OR = 2.86, 95% CI = 1.72-4.74, p < 0.001), and matching into neurosurgery (OR = 5.31, 95% CI = 2.78-9.46, p < 0.001) predicted H-index when applicants who had never published were removed (Table 4).

**Table 4:** Multivariate and univariate predictors of H-index. Univariate and multivariate analysis of factors associated with increased h index support the relationships shown in Figure 3. *: significant p-value; NIH: National Institutes of Health; OR: odds ratio; CI: confidence interval

Variable	Univariate OR/β (95% CI)	P-value	Multivariate OR/β (95% CI)	P-value
Top-40 NIH-funded medical school	2.32 (1.63, 3.31)	<0.001*	2.31 (1.62, 3.38)	<0.001*
Home program	1.36 (0.93, 1.99)	0.11	0.64 (0.42, 0.98)	0.04*
Additional degree	4.13 (2.51, 6.79)	<0.001*	2.86 (1.72, 4.74)	<0.001*
Specialty				
Orthopedic surgery	0.98 (0.63, 1.53)	0.92	1.12 (0.72, 1.75)	0.61
Vascular surgery	0.55 (0.21, 1.43)	0.22	0.75 (0.29, 1.96)	0.56
Otolaryngology	0.88 (0.67, 1.31)	0.51	0.84 (0.56, 1.27)	0.49
Plastic surgery	0.77 (0.44, 1.34)	0.35	0.71 (0.41, 1.23)	0.22
Neurosurgery	4.78 (2.74, 8.34)	<0.001*	5.13 (2.78, 9.46)	<0.001*

Discussion

Matched applicants in neurosurgery and plastic surgery have the highest median number of peer-reviewed publications, predict publication output, and are disproportionately represented among applicants with high research output, whereas matching into vascular surgery and orthopedic surgery correlates with the lowest median number of peer-reviewed publications and predicts applicants having never published. This trend aligns with the rank order of research output listed in the “Charting Outcomes of the Match” document released by the NRMP. However, this document does not group allopathic, osteopathic, and foreign medical graduates together, as we have. Additionally, our measure of research output was limited to peer-reviewed publications, whereas the “Charting Outcomes of the Match” document combines APPs, making direct comparisons challenging [1].

Data released by the NRMP indicates a consistent increase in research output across all specialties over time [1]. Following the USMLE Step 1 exam’s transition to a pass-fail grading system, several surveys have explored its impact on selection criteria within competitive specialties. Program directors in neurosurgery and plastic surgery have noted an increased emphasis on research, ranking it among the top factors for selection [5-9]. Directors from plastic surgery anticipate that students will increasingly take time off from medical school to pursue research [9]. However, surveys assessing factors for residency selection in orthopedic surgery, otolaryngology, and vascular surgery suggest that while the importance of research has grown, it is not a high priority [6-8]. This indicates that the emphasis on research output may not be as critical in residency selection for these specialties as it is in neurosurgery and plastic surgery, and our results provide further evidence of this.

When individuals with zero publications were excluded from the dataset, the difference between the median number of publications and the median H-index was most pronounced in neurosurgery. However, the H-index in plastic surgery and vascular surgery remained stable even after removing non-publishers, indicating a consistent impact of published research within these specialties. In contrast, in orthopedic surgery and otolaryngology, the H-index showed a notable increase once applicants without publications were excluded, suggesting that the research produced by those who do publish is cited more frequently. This divergence in publication impact points to variability, where, in certain specialties, a smaller cohort of researchers makes a more substantial contribution to the field’s scholarly impact. While the H-index offers insights into the impact of applicants’ publications, it is also influenced by field-specific citation practices and publication volume norms, and therefore, may not reflect research quality uniformly across specialties.

Attending a medical school ranked among the top-40 NIH-funded institutions was a predictor of publication volume and had a protective effect against never having published. The number of active researchers at an institution influences the NIH funding received, which, in turn, provides a greater number and variety of opportunities for students. Funds are often allocated to advanced equipment or supplies, along with research support staff, enhancing productivity [14].

Gaining an additional degree, beyond a medical degree, had a more substantial effect on publication volume than attending a top 40 NIH-funded institution, but did not significantly protect from never having published. These findings suggest that skills acquired outside the traditional medical school curriculum and time spent away from medical studies influence applicants’ research output, but do not guarantee academic productivity. More students are taking research years during medical school, and more medical schools are integrating dedicated research time into their curricula. Unsurprisingly, dedicated research time and education focused on statistics and methodology would enhance research output.

Given the substantial impact of obtaining an additional degree and the growing trend of taking gap years, program directors might consider these factors when evaluating how applicants capitalize on opportunity. While additional education increases academic productivity and may allow students from less prestigious medical schools to enhance the research portion of their application, additional education and gap years not only extend an already lengthy career path but also vary in feasibility due to applicants’ financial situations. Both pursuing additional degrees and taking gap years require funding and sacrificing potential earnings, thus impacting the financial trajectories of future physicians. This disproportionate burden may dissuade otherwise qualified candidates from less advantaged backgrounds, thus limiting diversity in competitive surgical specialties.

The influence of medical school funding, completion of additional degrees, and specialty match can contribute to possible inequities, particularly in neurosurgery and plastic surgery. Most medical students do not attend top-40 NIH-funded institutions, and their diverse financial backgrounds affect their ability to pursue additional degrees or engage in research years. As these factors significantly influence publication volume, students from less prestigious schools or those unable to afford extra academic years may be disadvantaged. Understanding what drives publication volume is crucial for a fair assessment of applicants’ academic productivity. It is vital to consider students’ access to resources and opportunities when evaluating their qualifications, ensuring fairness as research importance increases in residency selection. These disparities may reflect broader systemic factors related to access, mentorship, and institutional infrastructure that shape research opportunities across socioeconomic and institutional backgrounds.

Limitations and future directions

Information concerning unmatched applicants is not publicly available. The NRMP releases differences in the mean number of APPs between matched and unmatched applicants; however, both the distribution and number of peer-reviewed publications are unlisted. Therefore, the influence of peer-reviewed publications on matching into competitive surgical residency programs cannot be determined via NRMP data.

Numerous factors influence match outcomes besides research. Research is just one component among many, with survey data from program directors indicating that USMLE Step 2 scores, letters of recommendation, sub-internship performance, and perceived “fit” within the program, among others, also play a significant role in interview selection and the final ranking for residency positions [5-9]. Among these influential factors, research is the only metric publicly accessible. Information on USMLE Step 2 scores, letters of recommendation, sub-internship performance, and perceived “fit” within the program cannot be accessed outside of an institution’s historical records of residency applicants, and no institution receives applications from every applicant in their specialty. However, this information is unlikely to become available.

Additionally, the influence of academic productivity on the NRMP match is not deterministic. We have shown that applicants who have never published can still match into competitive surgical specialties, although the likelihood varies by specialty. Furthermore, survey data from program directors indicate that research is not the most valued criterion for residency selection. Features consistently rated as important by program directors, which are not publicly available, include USMLE Step 2 scores, letters of recommendation, sub-internship performance, and perceived “fit” within the program, among others [5-9].

Additional limitations include potential selection and classification biases introduced during data collection and adjudication, particularly in assigning specialty-specific publications. While efforts were made to verify author identity and publication type, inaccuracies may persist due to common names or incomplete affiliations. Additionally, findings may not be generalizable to unmatched applicants or international residency systems, as our data is restricted to U.S.-matched candidates.

Lastly, as a retrospective observational study, our results describe associations only and should not be interpreted as causal. Because the dataset includes only matched applicants from U.S. residency programs, findings may not generalize to international or unmatched applicants.

## Conclusions

This analysis highlights distinctions in academic productivity across specialties and examines the impact of graduating from a top-40 NIH-funded medical school and obtaining an additional non-medical degree on publication volume. Neurosurgery and plastic surgery have the highest median number of publications. Differences are also noted in the percentage of matched applicants who have never published and the percentage of papers that are cited (via the H-index). Our findings suggest that disparities in perceived medical school prestige, financial limitations affecting the ability to pursue additional degrees or research years, and varying publication expectations across specialties are all probable sources of inequities in competitive residency matching. To mitigate these disparities, we recommend expanding funded research opportunities for students at institutions with fewer resources, encouraging residency programs to consider applicants’ access to research infrastructure when evaluating publication metrics, and supporting initiatives that promote transparency and standardization in how research productivity is assessed during the residency selection process.
